# Unusually Effective Blue-to-UVC Upconversion of Pr^3+^-Doped Sr_3_Lu(PO_4_)_3_ and Ba_3_Lu(PO_4_)_3_ Phosphors: A Comparative Study

**DOI:** 10.1021/acs.inorgchem.5c01458

**Published:** 2025-05-29

**Authors:** Nadiia Rebrova, Alexander Grippa, Patrycja Zdeb-Stańczykowska, Przemysław J. Dereń

**Affiliations:** † 215275 Institute of Low Temperature and Structure Research, Polish Academy of Science, ul. Okólna 2, Wrocław 50-422, Poland; ‡ Institute for Scintillation Materials, National Academy of Sciences of Ukraine, Nauky Avenue, 60, Kharkiv 61001, Ukraine

## Abstract

Many pathogens, including bacteria and viruses, are increasingly developing resistance to conventional disinfectants. As a result, new approaches to disinfection are being explored, such as the use luminophores that convert visible light into ultraviolet C radiation (UVC). In this work, we present novel UVC phosphors, A_3_Lu­(PO_4_)_3_ (A = Sr, Ba), activated by Pr^3+^. These phosphates were synthesized by the Pechini method with different activator concentrations and crystallized in a cubic structure with the space group *I4̅3d*. The emission and excitation spectra, as well as decay times under synchrotron and visible-light excitation, were measured. Both phosphates exhibited efficient and fast 5d–4f emission from 240 to 340 nm, along with a very weak f-f emission line around 600 nm upon ultraviolet excitation. Multiphonon relaxation from the ^3^P_0_ to ^1^D_2_ state causes the phosphors to emit weak blue (^3^P_1_ → ^3^H_4_) and intense orange (^1^D_2_ → ^3^H_4_) light upon direct excitation of the ^3^P_2_ level. The upconversion properties of A_3_Lu­(PO_4_)_3_:Pr^3+^ crystallites were investigated under 444 nm laser excitation, and the effect of Pr^3+^ concentration on these properties was evaluated. Compared to the YPO_4_:Pr^3+^ reference material studied earlier, the Ba_3_Lu­(PO_4_)_3_:Pr^3+^ host showed a 20-fold enhancement in UVC upconversion emission, making this phosphate one of the most efficient visible-to-ultraviolet upconversion matrices to date. Thus, the studied phosphates have potential use in sterilization, disinfection, photocatalysis, and phototherapy.

## Graphical abstract

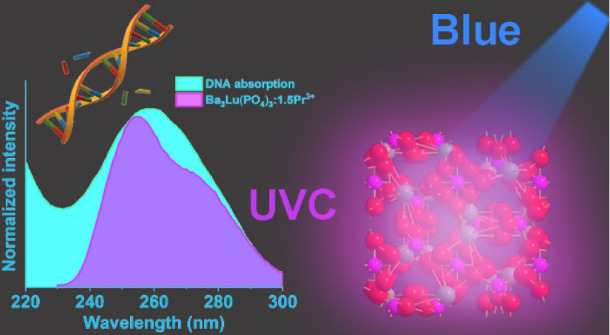

## 1 Introduction

Inorganic compounds doped with trivalent lanthanide ions are widely used in various advanced optical and electronic applications due to their sharp emission lines and excellent thermal and chemical stability. Typical applications include phosphors for light-emitting diodes (LED), display technologies, bioimaging and medical diagnostics, anticounterfeiting measures, laser materials, radiation detectors, and dosimeters.^1–4^

Recently, researchers have increasingly focused on a unique process known as upconversion in which multiple low-energy photons are combined to produce a single high-energy photon.^5^ This property opens a wide range of applications, especially in biomedical imaging, photodynamic therapy, solar energy harvesting, and disinfection.^6–10^ In the latter case, phosphors are being developed that can convert visible light into ultraviolet-C (UV–C) radiation, which has a cytotoxic effect on Deoxyribonucleic Acid (DNA) and ribonucleic acid (RNA), ultimately leading to apoptosis of microorganisms. This development aims to replace mercury lamps with more environmentally friendly and efficient materials. Praseodymium ion (Pr^3+^) is commonly used as an activator because, depending on the host, it can exhibit efficient photoluminescence in the UV–C range due to the interconfiguration transitions 4f5d → 4f. Ideal matrices for upconversion are compounds in which the ^1^S_0_ level of the praseodymium ion is significantly above the lower edge of the 4f5d configuration.^11^ Currently, UVC upconversion of Pr^3+^ ions excited by visible light is being studied in various matrices, including fluorides (RbCaF_3_, LiYF_4_), phosphates (YPO_4_), oxyfluorides (Y_7_O_6_O_9_, Lu_7_O_6_F_9_, LuOBr), borates (YBO_3_) and silicates (Y_2_SiO_5_, LiY_9_(SiO_4_)_6_O_2_).^12–19^ Due to their simple preparation process, excellent chemical stability, and radiation resistance, phosphates are ideal candidates for upconversion studies.

Furthermore, in our previous work on phosphate upconversion, the praseodymium-activated compound Ba_3_Y­(PO_4_)_3_ showed a 6-fold increase in upconversion emission compared to the known YPO_4_:Pr^3+^. We decided to build on this success, and in this paper we focus on the upconversion properties of Pr^3+^ -doped eulytite-type orthophosphates A_3_Lu­(PO_4_)_3_ (A = Sr, Ba). These compounds, doped with rare earth metals such as Ce^3+^, Eu^3+^, Sm^3+^, and Dy^3+^, have been extensively studied as suitable matrices for various potential optical applications, including displays and LEDs.^20–27^ To our knowledge, there is currently only one study in the literature on time-resolved vacuum ultraviolet (VUV) spectroscopy of Ba_3_Lu­(PO_4_)_3_:Pr^3+^ using synchrotron radiation excitation, and nothing has been reported for strontium-based phosphate.^28^ The observed 5d-4f emission of Pr^3+^ ions in the ultraviolet region confirms the potential of A_3_Lu­(PO_4_)_3_ (A = Sr, Ba) hosts to convert visible light to the UVC region of the spectrum. For the first time, visible luminescence and upconversion properties, including upconversion emission intensity, decay time, and the dependence of emission on pump power, were measured for A_3_Lu­(PO_4_)_3_:Pr^3+^ (A = Sr, Ba) crystallites. The study examined the effect of activator concentration on upconversion properties, allowing for the determination of the optimal concentration. When excited by a 444 nm laser, barium-containing phosphate produces ultraviolet emission that is 20 times more intense than that of standard YPO_4_:Pr^3+^, making it promising for practical applications.

## 2 Experiment

Powder samples of A_3_Lu_1–*x*_Pr_*x*_(PO_4_)_3_ (A = Sr, Ba) with x = 0, 0.001, 0.005, 0.01, 0.015, and 0.02 were synthesized using the Pechini method. Lutetium oxide (Lu_2_O_3_; 99.9%), strontium carbonate (SrCO_3_; 99.9%), barium acetate (BaCO_3_; 99.9%), ammonium dihydrogen phosphate (NH_4_H_2_PO_4_; 99.99%), praseodymium oxide (Pr_2_O_3_; 99.9%), citric acid (C_6_H_8_O_7_, 99.7%) and ethylene glycol (EG, C_2_H_4_(OH)_2_, 99%) were used as raw materials without further purification. Stoichiometric amounts of Lu_2_O_3_, ACO_3_, and Pr_2_O_3_ were dissolved in nitric acid with stirring and heating. To the resulting transparent solution, maintained at room temperature under vigorous stirring, solutions of citric acid, NH_4_H_2_PO_4_, and ethylene glycol were added successively. A ratio of citric acid, ethylene glycol and metal cations of 2:2:1 was used. The solution was heated on a hot plate at 80 °C for 24 h to produce a white resin, which was then placed in a crucible. The resin was heat treated in two stages: at 500 °C for 6 h and at 1250 °C for 5 h. Figure 1 schematically shows the stages of the preparation of A_3_Lu­(PO_4_)_3_:Pr^3+^ (A = Sr, Ba) crystallites.

**Figure 1:**
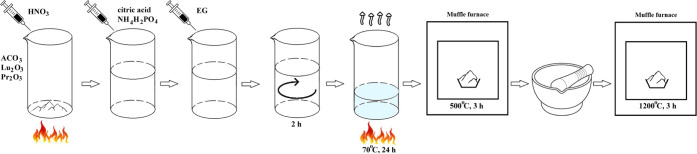
Schematic illustration of the synthesis of A_3_Lu­(PO_4_)_3_:Pr^3+^ (A = Sr, Ba) crystallites.

The phase purity of the A_3_Lu­(PO_4_)_3_:Pr^3+^ (A = Sr, Ba) crystallites was checked on an X’Pert PRO X-ray diffractometer with CuKα radiation (λ = 1.54056 Å). The morphology of A_3_Lu­(PO_4_)_3_:Pr^3+^ (A = Sr, Ba) was examined using a FEI Nova NanoSEM 230 field emission scanning electron microscope (FE-SEM). Images were acquired at 5.0 kV in slow scan mode to enhance resolution and contrast. The powder sample was embedded in carbon resin prior to analysis. Excitation and emission spectra, as well as luminescent decay time, were measured on an FLS1000 fluorescence spectrometer equipped with a 450 W ozone-free xenon lamp and a xenon flash lamp. Upconversion measurements were performed using a VUV McPherson spectrometer equipped with a Hamamatsu R7154P photomultiplier, a UG5 filter (Eksma Optics), and 444 nm continuous-wave laser excitation. To study the upconversion lifetime, the second harmonic of a titanium-sapphire laser, pumped by the second harmonic of a Nd:YAG laser (LOTIS TII Belarus), was used.

The study of excitation and emission spectra, as well as decay kinetics in the ultraviolet range, was conducted at the SUPERLUMI station in DESY (Hamburg, Germany) using a 2-m McPherson monochromator at a synchrotron radiation source. A Hamamatsu R6358 photomultiplier was used as the detector. The excitation spectra were corrected for the incident photon flux of the excitation beam using the excitation spectrum of a reference sodium salicylate sample. The emission spectra were corrected to account for the sensitivity of the detection system.

## 3 Results and Discussion

### 3.1 Synthesis and Characterization of the Structure

The obtained Sr_3_Lu­(PO4)_3_:Pr^3+^ and Ba_3_Lu­(PO_4_)_3_:Pr^3+^ phosphor powders were assigned to the cubic structure with the space group *I*4*®3d* (Figure 2 a,b). The samples completely crystallized without forming any secondary phases. Although the phosphates were synthesized using the Pechini method, the resulting particles are highly agglomerated, with sizes ranging from a few microns to several tens of microns (Figure S1). Figure 2c,d shows a visualization of the phosphate unit cell and the coordination polyhedra of the A^2+^/Lu^3+^ cation (A = Ba, Sr). Taking into account the influence of valence, it is assumed that Pr^3+^ more readily replaces Lu^3+^ ions in the crystal lattice compared to divalent alkaline earth metal ions. In the structure, the A^2+^ and Lu^3+^ cations randomly occupy the same *C*_3_ symmetry site, with site occupancies of 0.75 and 0.25, respectively.^29^ Since the oxygen atoms are also randomly located at two C_1_ sites, there is significant disorder in the crystal lattice, which is reflected in the emission spectra described later in this paper.

**Figure 2:**
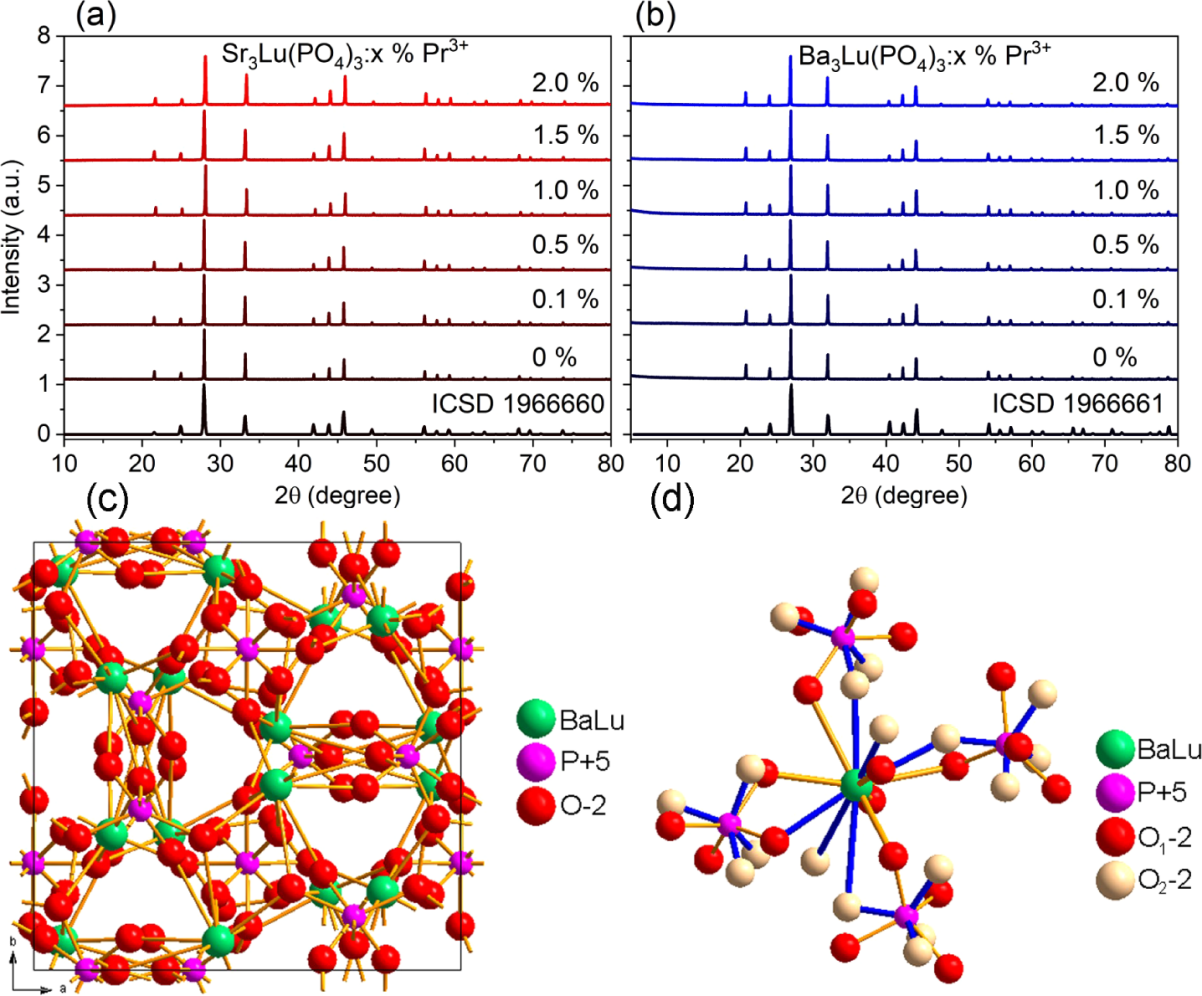
XRPD patterns of Sr_3_Lu­(PO_4_)_3_:Pr^3+^ (a) and Ba_3_Lu­(PO_4_)_3_:Pr^3+^ (b) phosphors. Crystal structure of A_3_Lu­(PO_4_)_3_ (A = Sr, Ba) (c) and coordination of A^2+^/Lu^3+^ cation (d).

### 3.2 Luminescence Properties in the Visible Range

Figure 3 a,c shows the excitation spectra of the A_3_Lu­(PO_4_)_3_:0.5%Pr^3+^ (A = Sr, Ba) phosphors, with measurements taken at 620 nm. Two excitation bands, located in the spectral regions of 425–500 nm and 560–610 nm, correspond to the transition from the ground state of Pr^3+^ (^3^H_4_) to ^3^P_J_ and ^1^D_2_, respectively. Since the host lattice is disordered and the Pr^3+^ ions are located in sites that differ in the crystal field strength, the measured excitation bands consist of relatively structureless and significantly broadened bands. This makes these phosphates promising for applications such as the conversion of sunlight to ultraviolet light.

**Figure 3:**
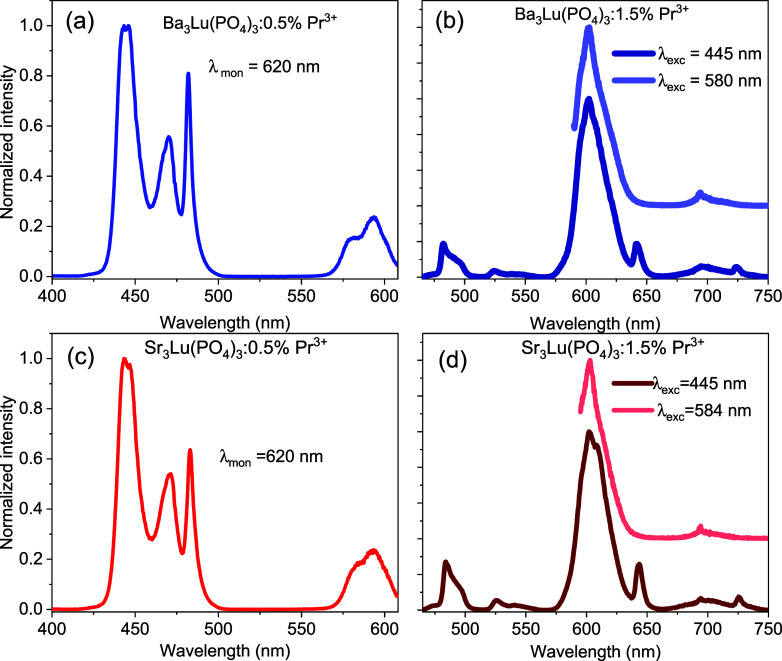
Excitation (a, c) and emission (b, d) spectra of the A_3_Lu­(PO_4_)_3_:0.5%Pr^3+^ (A = Sr, Ba) phosphors.

Figure 3b,d shows typical emission spectra of Pr^3+^ ions in phosphate matrices when excited at a wavelength of 445 nm. Since the ^3^P_2_, ^3^P_1_ and ^3^P_0_ levels are very close to each other, the excitation of ^3^P_2_ (445 nm) leads to rapid nonradiative relaxation to the ^3^P_1_ and ^3^P_0_ states. Then, the excited ^3^P_0_ state is depopulated into ^1^D_2_ via multiphonon relaxation due to the high phonon energy in the phosphate matrices, which is around 1200 cm^–1^.^30,31^ The energy gap between ^3^P_0_ and ^1^D_2_ levels in the studied phosphates is ∼3500 cm^–1^, and about three phonons are required for relaxation from ^3^P_0_ to ^1^D_2_.^25^ Therefore, the emission from the ^1^D_2_→^3^H_4_ transition dominates the spectra, while the emission from the ^3^P_1,0_ to ^3^H_J_,^3^ F_J_ levels is weak. To confirm that the intense orange luminescence is due to the ^1^D_2_ → ^3^H_4_ transition, the luminescence was studied under direct excitation of the ^1^D_2_ level at 580 and 584 nm (Figure 3 b,d).

It should be noted that luminescence under ^3^P_0_ excitation strongly depends on the activator concentration. Specifically, as the concentration increases, emission in the 605–610 nm range decreases, likely due to cross-relaxation between neighboring optically active ions. For clarity, the spectra were normalized to the intensity at ∼482 nm (^3^P_0_→^3^H_4_) (Figure S2).

The decay of luminescence of A_3_Lu­(PO_4_)_3_:Pr^3+^ (A = Sr, Ba) phosphors from the ^3^P_0_ and ^1^D_2_ states was studied as a function of Pr^3+^ concentration (Figure 4). Since the decay curves of these emissions deviate from an exponential dependence, the luminescent lifetime was determined using the following equation:^32,33^

1 $$\tau =\frac{\int tI\left(t\right)dt}{\int I\left(t\right)dt}$$

**Figure 4:**
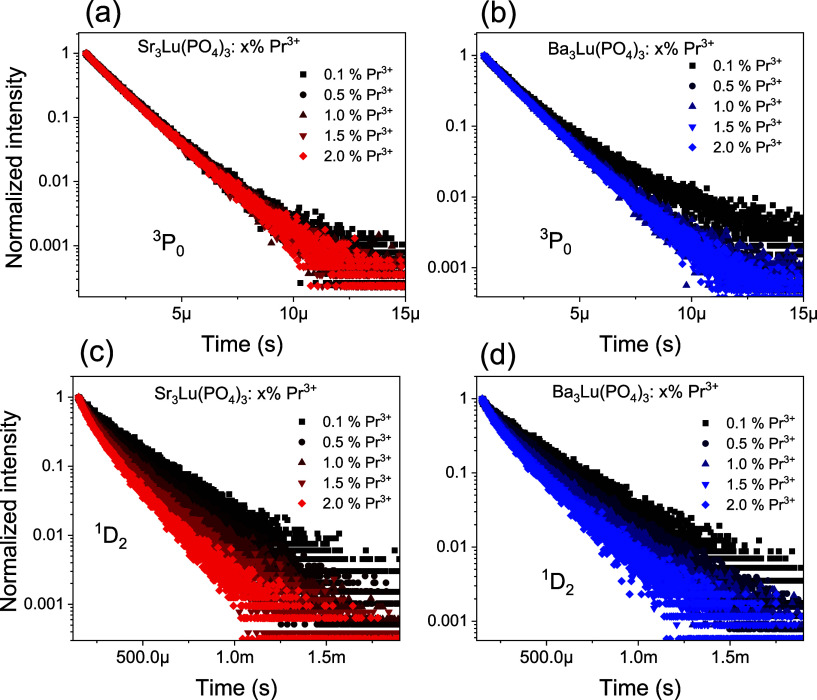
Luminescent decay curves of the ^3^P_0_ (a, b) and ^1^D_2_ (c, d) emission of the A_3_Lu­(PO_4_)_3_:Pr^3+^ (A = Sr, Ba) phosphors.

The integration of the emission intensity was performed for all samples on a scale from 1 to 0. Previously, all minima of the spectra were shifted to zero using the baseline determined for each spectrum separately. The nonexponential luminescence decay kinetics can be related to nonradiative energy transfer processes between excited and unexcited Pr^3+^ ions. Additionally, the variation in the local crystal field surrounding different Pr^3+^ ions causes slight shifts in energy levels and transition rates, leading to a distribution of decay times.^34^ The luminescence decay constants are presented in Table 1. Concentration quenching of luminescence causes a decrease in the decay constants with increasing concentration of praseodymium ions in the samples.^35,36^ Typically, in praseodymium-activated matrices, the ^1^D_2_ level decays faster with increasing activator concentration in the samples compared to the ^3^P_0_ level.^37^ This is due to the process of cross-relaxation, which is effective by being allowed by spin. Cross-relaxation occurs in Pr^3+^ ion pairs when the ions are close enough to each other, following the transition [^1^D_2_, ^3^H_4_] → [^1^G_4_, ^3^F_4_].^38^ Cross-relaxation is also effective in depopulating the ^3^P_0_ level, but only at room temperature, when the ^3^P_0_ is thermally excited through the [^3^P_0_, ^3^H_4_] → [^1^D_2_, ^3^H_6_] transition. Note that the strontium-based phosphate exhibited a slightly faster luminescence decay constant compared to that of its barium-based counterpart. This is confirmed by the more pronounced cross-relaxation for Sr_3_Lu­(PO_4_)_3_:Pr^3+^ (Figure S3).

**Table 1:** Luminescent Lifetime for the ^3^P_0_ and ^1^D_2_ Transition of Pr^3+^ in A_3_Lu­(PO_4_)_3_ (A = Sr, Ba) Phosphors

	Sr_3_Lu(PO_4_)_3_:Pr^3+^		Ba_3_Lu(PO_4_)_3_:Pr^3+^	
Concentration Pr^3+^, mol %	^3^P_0_ [μs]	^1^D_2_ [μs]	^3^P_0_ [μs]	^1^D_2_ [μs]
0.1	2.15	375	2.65	408
0.5	2.13	355	2.24	368
1.0	2.10	320	2.21	344
1.5	2.09	294	2.17	319
2.0	2.08	275	2.16	313

### 3.3 Luminescence Properties in the Ultraviolet Range

#### 3.3.1 Stokes Emission

Using synchrotron radiation, the emission and excitation spectra of Pr^3+^-doped and undoped matrices were studied at 8 K (Figure 5). The excitation spectra of Ba_3_Lu­(PO_4_)_3_:0.5% Pr^3+^ and Sr_3_Lu­(PO_4_)_3_:0.5% Pr^3+^, monitored at 280 and 275 nm, can be attributed to the 4f^2^→4f^1^5d^1^ excitation transitions of Pr^3+^. Figure 5a,c shows the emission spectra of Ba_3_Lu­(PO_4_)_3_:0.5% Pr^3+^ and Sr_3_Lu­(PO_4_)_3_:0.5%Pr^3+^ obtained upon excitation of Pr^3+^ 4f^2^ → 4f^1^5d^1^ at 190 and 205 nm, respectively. The emission bands detected in the 240 to 340 nm range for both compounds are associated with interconfigurational transitions originating from the lowest excited 4f^1^5d^1^ state of Pr^3+^ and terminating at the ^3^H_J_ and ^3^F_J_ multiplets of the 4f^2^ ground-state electronic configuration. This is confirmed by the single-exponential behavior of this emission, with an approximate lifetime of τ ≈ 14 ns (Figure 6a). The luminescence spectrum of Ba_3_Lu­(PO_4_)_3_:0.5% Pr^3+^ exhibits a broad band with a maximum at 374 nm, which is attributed to the emission from its intrinsic defects.^39,40^ The lifetime of this emission is 0.5 ns when excited at 190 nm at 8 K (Figure 6b). No impurity luminescence is observed in the emission spectrum of Sr_3_Lu­(PO_4_)_3_:0.5%Pr^3+^, as the sample was excited using a wavelength of lower excitation energy (205 nm) compared to the barium-containing compound (190 nm). However, time-resolved emission spectroscopy studies with 190 nm excitation confirm the presence of impurity luminescence characterized by a fast decay constant of 0.4 ns (Figure 6b). Under deep ultraviolet excitation, the emission spectra for both undoped hosts exhibit a broad band ranging from 250 to 500 nm, attributed to intrinsic luminescence caused by self-trapped excitons in pure matrices (Figure 5b,d). Similar emission is also observed in other undoped compounds.^41–43^ The excitation spectra of both undoped phosphors correspond to their exciton absorption spectra.^42^

**Figure 5:**
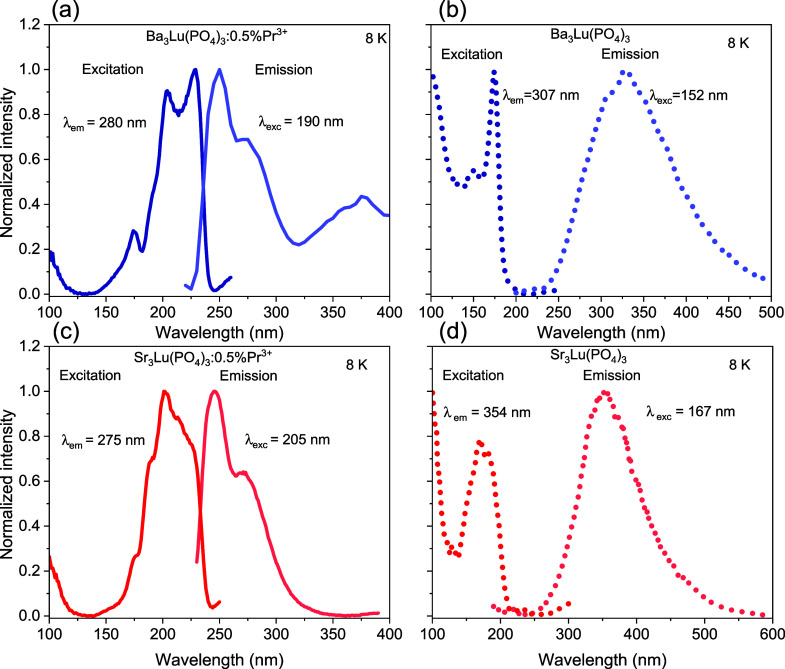
Excitation and emission spectra of Ba_3_Lu­(PO_4_)_3_:0.5% Pr^3+^ (a), Ba_3_Lu­(PO_4_)_3_ (b), Sr_3_Lu­(PO_4_)_3_:0.5% Pr^3+^ (c), and Sr_3_Lu­(PO_4_)_3_ (d) using synchrotron radiation.

**Figure 6:**
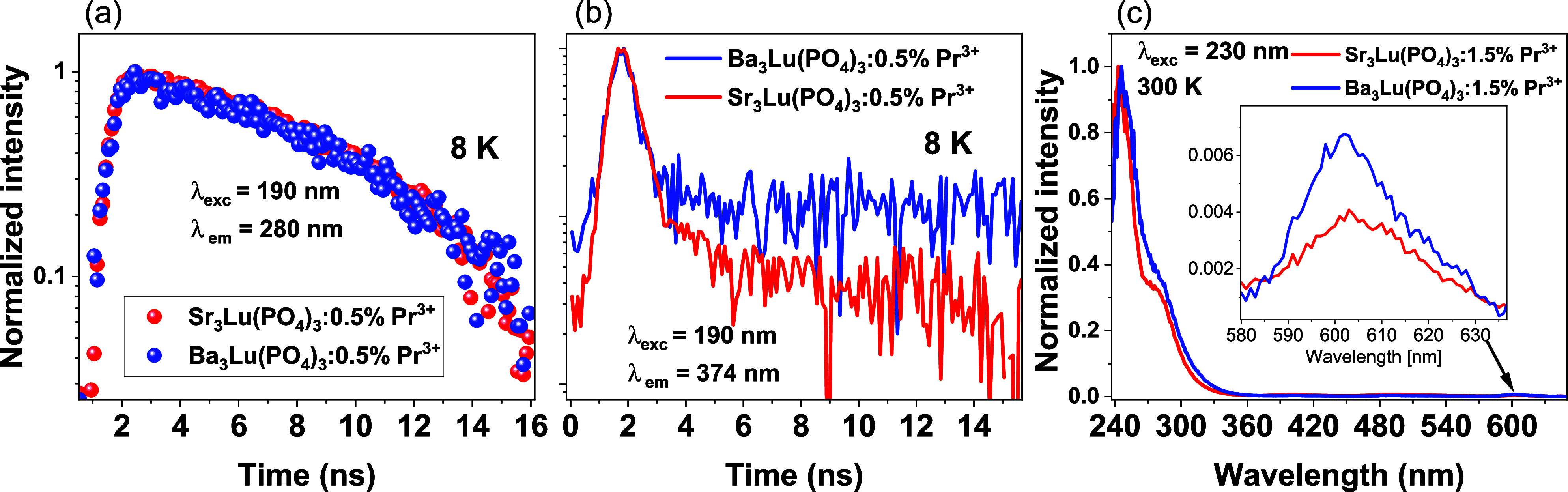
Decay times of A_3_Lu­(PO_4_)_3_:0.5% Pr^3+^ (A = Sr, Ba) under 190 nm excitation, monitored at 280 nm (a) and 374 nm (b). (c) Room-temperature luminescence spectra of Sr_3_Lu­(PO_4_)_3_:1.5%Pr^3+^ and Ba_3_Lu­(PO_4_)_3_:1.5% Pr^3+^ under 230 nm excitation.

Based on the excitation and emission spectra, the Stokes shift of Ba_3_Lu­(PO_4_)_3_:0.5% Pr^3+^ is 1463 cm^–1^, while for Sr_3_Lu­(PO_4_)_3_:0.5%Pr^3+^ it is 1519 cm^–1^. The Stokes shift (ΔS) was calculated as the energy difference between the position of the lowest energy excitation band and the highest energy emission band (E_em_).^44^ The lowest 4f^1^5d^1^ energy level for Pr^3+^ (E_fd_) for crystallites was estimated using the equations:^13^

2 $$E_{fd}=E_{em}+1/2\Delta S$$

The lowest 4f^1^5d^1^ energy level for Ba_3_Lu­(PO_4_)_3_:0.5% Pr^3+^ is 42,520 cm^–1^, and for Sr_3_Lu­(PO_4_)_3_:0.5%Pr^3+^ it is 42,780 cm^–1^. These values will help later determine which levels of praseodymium are involved in the upconversion process.

Due to the small Stokes shift, the excited 5d electronic configuration depopulates radiatively without feeding the ^3^P_J_ term of the 4f electronic configuration. This is confirmed by the negligible emission in the visible range (Figure 6c). The very weak band around 600 nm is associated with the intraconfigurational 4f^2^ → 4f^2^ transitions of Pr^3+^ from the ^1^D_2_ level to the ^3^H_4_ ground state, resulting from nonradiative energy transfer from 5d4f to 4f^2^. The spectra show the absence of ^3^P_0_ emission, which is quenched by multiphonon relaxation to the ^1^D_2_ multiplet, as observed in the case of Ca_9_Lu­(PO_4_)_7_:Pr^3+^ and LaPO_4_:Pr^3+^ phosphates.^44–46^

#### 3.3.2 Anti-Stokes Emission

The upconversion emission spectra of A_3_Lu­(PO_4_)_3_:1.5% Pr^3+^ (A = Sr, Ba) phosphors excited by a 444 nm laser are shown in Figure 7a. The phosphors emit ultraviolet emission in the range of 235 to 315 nm, with approximately 85% of the total intensity falling in the UVC range and 15% in the UVB range. The integrated upconversion intensity of the studied phosphates in the UVC region was compared with the previously published effective upconverter YPO_4_:Pr^3+^.^17^ The studies were carried out under identical conditions, with Sr_3_Lu­(PO_4_)_3_:1.5% Pr^3+^ showing a 4-fold increase and Ba_3_Lu­(PO_4_)_3_:1.5% Pr^3+^ a 20-fold increase. Furthermore, the barium-containing phosphate exhibited an almost 4-fold increase in upconversion luminescence in the UVC region compared to Y_2_SiO_5_:Pr^3+^. To evaluate the potential applicability of newly synthesized phosphors, the upconversion luminescence spectra were normalized to the absorption spectra of DNA (black line), a mercury lamp (pink line) and the shortest wavelength UV LED currently available (blue line) (Figure 7b).^47–49^ The near-complete overlap between the upconversion luminescence and DNA absorption spectrum suggests that DNA may absorb UVC radiation emitted by the phosphor, potentially leading to DNA denaturation.

**Figure 7:**
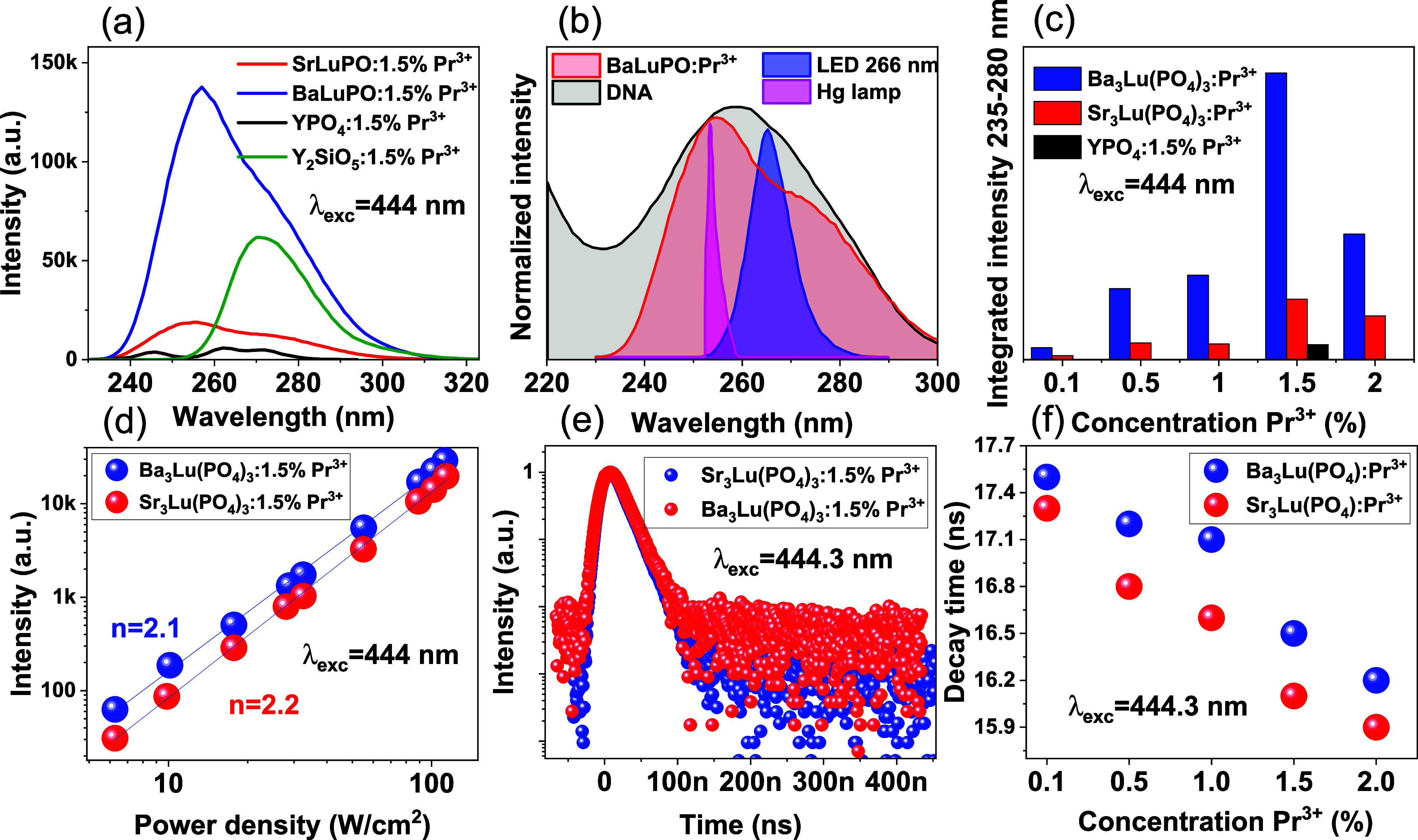
(a) Upconversion emission spectra of A_3_Lu­(PO_4_)_3_:1.5% Pr^3+^ (A = Sr, Ba), YPO_4_:1.5% Pr^3+^, and Y_2_SiO_5_:1.5% Pr^3+^ phosphors excited by a 444 nm laser. (b) Normalized typical absorption spectra of DNA, combined with the spectra of UVC LED, mercury (Hg) lamp and upconversion emission Ba_3_Lu­(PO_4_)_3_:1.5Pr^3+^ under laser excitation 444 nm. (c) Concentration of Pr^3+^ versus integrated upconversion emission intensity in the 235–280 nm range. (d) Dependence of the intensity of upconversion emission on laser pump power for A_3_Lu­(PO_4_)_3_:1.5% Pr^3+^ phosphor. (e) Decay profiles of the upconverted luminescence of A_3_Lu­(PO_4_)_3_:1.5%Pr^3+^. (f) Concentration dependence of decay times for A_3_Lu­(PO_4_)_3_:Pr^3+^ (A = Sr, Ba).

The relationship between the intensity of upconversion and the Pr^3+^ concentration was also examined (Figure 7c). The emission intensity increased with the Pr^3+^ concentration, achieving optimal performance at 1.5 mol %. In compounds with high phonon energy and efficient energy transfer from the ^3^P_0_ to the ^1^D_2_ level as a result of multiphonon relaxation, the ^1^D_2_ multiplet can act as an intermediate level in the upconversion process.^50,51^ Taking the excitation wavelength of 444 nm (22,522 cm^–1^) and the ^1^D_2_ positions for Ba_3_Lu­(PO_4_)_3_:Pr^3+^ (∼17,250 cm^–1^) and Sr_3_Lu­(PO_4_)_3_:Pr^3+^ (∼17,200 cm^–1^), the ^1^D_2_ → 4f^1^5d^1^ transition does not contribute to the upconversion process, since such excitation terminates well below the lower limit of the 4f^1^5d^1^ band.

To gain a deeper understanding of the upconversion mechanism, the upconversion emission was measured at varying laser pump powers.^52^ For a fixed emission wavelength, the upconversion emission intensity (I) versus the laser pump power (P) provides information regarding the number of photons (n) contributing to the upconversion luminescence process. This proportionality relationship is given below:^53^

3 $$I\propto P^{n}$$

The graphs show a linear dependence of the upconversion emission intensity at a wavelength of 255 nm for Sr_3_Lu­(PO_4_)_3_:1.5% Pr^3+^ and 256 nm for Ba_3_Lu­(PO_4_)_3_:1.5% Pr^3+^, with laser pump powers in the range of 50–850 mW (Figure 7d). The slope values (n) were found to be 2.2 and 2.1, respectively, indicating that a two-photon process is responsible for the upconversion. Two two-photon mechanisms have been proposed in the literature to be responsible for the upconversion of praseodymium ions: energy transfer upconversion (ETU) and excited state absorption (ESA).^1^ Since these two processes are difficult to distinguish directly, upconversion lifetimes under pulsed excitation at a wavelength of 444.3 nm were studied to establish the mechanism in the phosphates (Figure 7e). The upconversion decay time constant (τ) was estimated using the equilibrium equation No 1. Since the upconversion lifetime falls within the range of 5d-4f emission decay kinetics, specifically 15.9–17.5 ns, the main mechanism responsible for the upconversion in the studied phosphates is ESA (Figure 7 f).^14^ For the ETU mechanism, the upconversion lifetime for our phosphates would be half the time of ^3^P_0_, approximately one microsecond (Table 1).

## 4 Conclusions

We successfully prepared Pr^3+^-doped A_3_Lu­(PO_4_)_3_ crystallites using the Pechini method. Under ultraviolet excitation, the phosphors exhibited an intense and fast emission band in the range of 240–340 nm, attributed to the 4f^1^5d^1^→4f^2^ interconfigurational transitions of Pr^3+^, along with a very weak emission line near 600 nm due to nonradiative energy transfer from 5d4f to 4f^2^. A_3_Lu­(PO_4_)_3_:Pr^3+^ phosphors emit orange light and show anti-Stokes emission in the germicidal region upon direct excitation of the ^3^P_2_ level. By examining the relationship between upconversion emission and activator concentration, we identified the optimal activator concentration to be 1.5 mol % for both compounds. Upconversion emission lifetime studies indicated that upconversion occurs as a result of Excited State Absorption. Ba_3_Lu­(PO_4_)_3_:Pr^3+^ crystallites exhibit highly efficient blue-to-UVC upconversion properties, which makes them attractive for antimicrobial applications. In addition, our research results indicate that the new phosphors exhibit UVC emission when exposed to ultraviolet light.

## Supplementary Material



## Notes

Figures. SEM images of Sr_3_Lu­(PO_4_)_3_:1.5Pr^3+^ and Ba_3_Lu­(PO_4_)_3_:1.5Pr^3+^ crystallites. The emission spectra of Sr_3_Lu­(PO_4_)_3_:xPr^3+^ and Ba_3_Lu­(PO_4_)_3_:xPr^3+^ crystallites under 445 nm excitation. Integral ratio of the ^3^P_0_→^3^H_4_ transition to the ^1^D_2_→^3^H_4_ transition (PDF)
