# Supplementary material for: Unusually Effective Blue-to-UVC Upconversion of Pr3+-Doped Sr3Lu(PO4)3 and Ba3Lu(PO4)3 Phosphors: A Comparative Study
Source: Inorg Chem. 2025 May 29;64(22):11146–54. doi: 10.1021/acs.inorgchem.5c01458 (PMC12152949; doi:10.1021/acs.inorgchem.5c01458)
Supplement: Supplementary file 1 [file ic5c01458_si_001.pdf]

Unusually Effective Blue-to-UVC Upconversion of Pr^{3+}-doped $Sr_3Lu(PO_4)_3$ and $Ba_3Lu(PO_4)_3$ Phosphors: A Comparative Study.

Nadiia Rebrova[a], Alexander Grippa[b] Patrycja Zdeb-Stańczykowska[a], and Przemysław J. Dereń[a]*

a Institute of Low Temperature and Structure Research, Polish Academy of Science, ul. Okólna 2, 50-422 Wrocław, Poland.

b Institute for Scintillation Materials, National Academy of Sciences of Ukraine, Nauky Avenue, 60, 61001, Kharkiv, Ukraine.

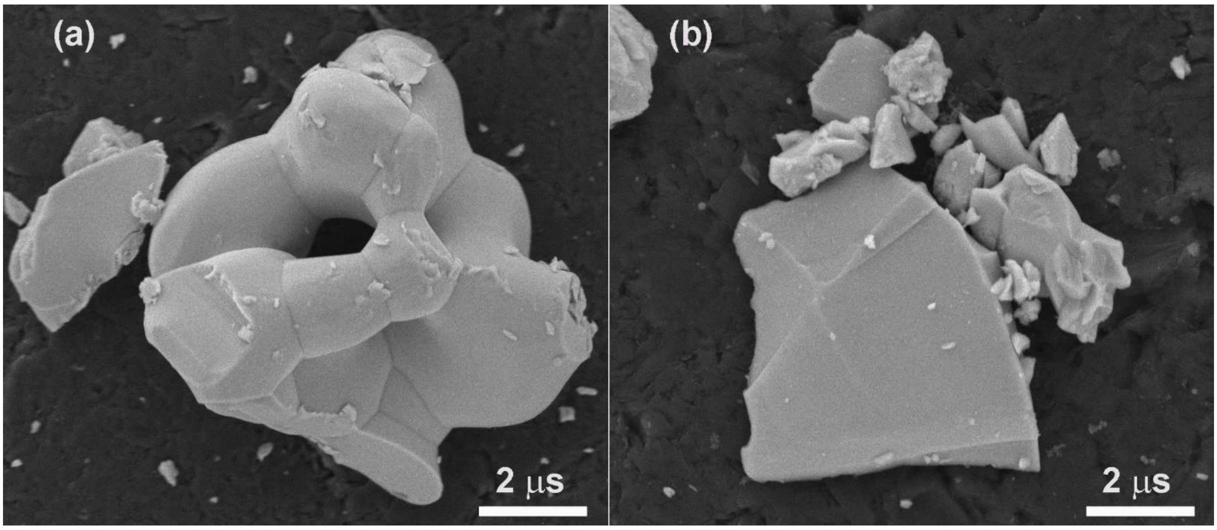

Figure S1. SEM images of $Sr_3Lu(PO_4)_3$:1.5Pr^{3+} (a) and $Ba_3Lu(PO_4)_3$:1.5Pr^{3+} (b) crystallites.

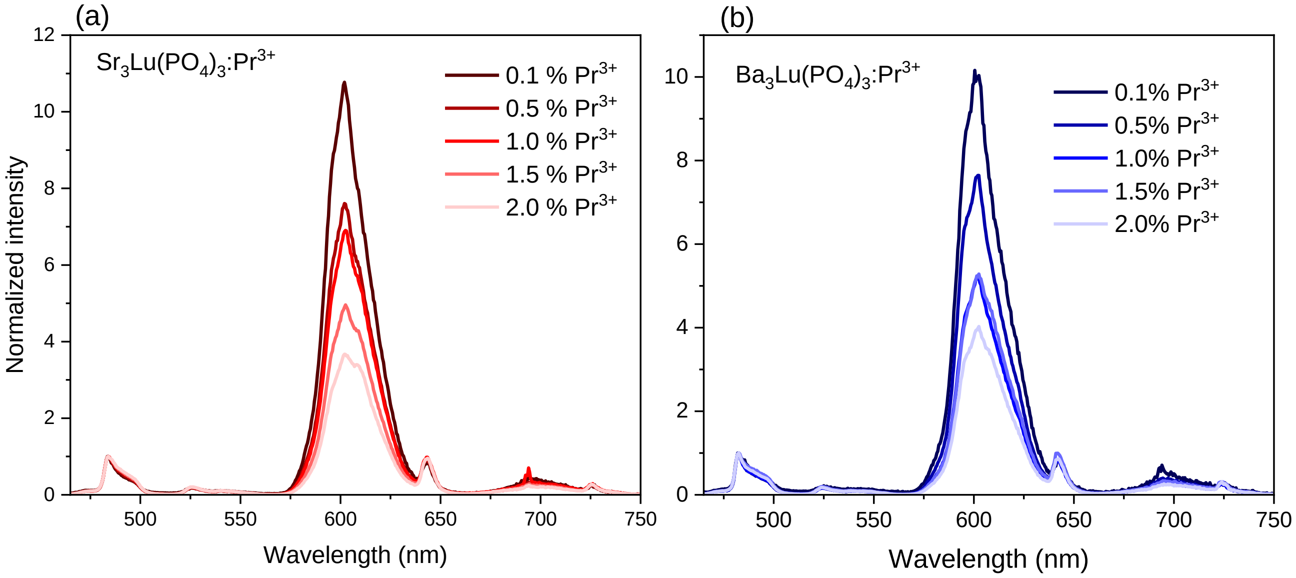

Figure S2. The emission spectra of $Sr_3Lu(PO_4)_3$:xPr^{3+} (a) and $Ba_3Lu(PO_4)_3$:xPr^{3+} (b) crystallites under 445 nm excitation. The emission spectra were normalized to the intensity at ~482 nm.

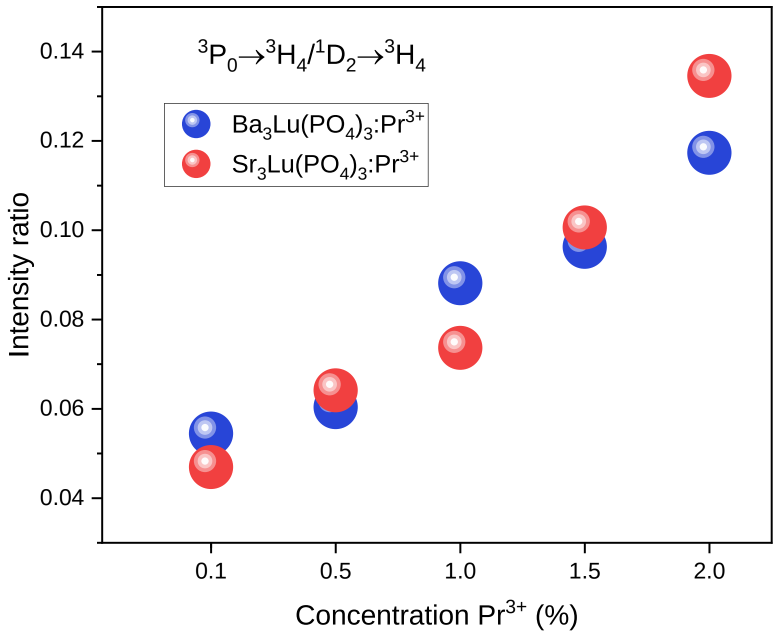

Figure S3. Integral ratio of the $^3P_0\rightarrow{}^3H_4$ transition to the $^1D_2\rightarrow{}^3H_4$ transition.
